# Screening African rice (*Oryza glaberrima)* for tolerance to abiotic stresses: I. Fe toxicity

**DOI:** 10.1016/j.fcr.2016.04.016

**Published:** 2018-05-01

**Authors:** M. Sikirou, A. Shittu, K.A. Konaté, A.T. Maji, A.S. Ngaujah, K.A. Sanni, S.A. Ogunbayo, I. Akintayo, K. Saito, K.N. Dramé, A. Ahanchédé, R. Venuprasad

**Affiliations:** a Laboratoire de Biologie Végétale, Département de Production Végétale, Faculté des Sciences Agronomiques, Université d'Abomey-Calavi, 01 BP 526, Cotonou, Benin; b Africa Rice Center (AfricaRice), 01 BP 2031, Cotonou, Benin; c Africa Rice Center (AfricaRice), c/o IITA, PMB 5320, Ibadan, Nigeria; d Institut national de l’environnement et de recherches agricoles (INERA), 01 B.P., 476, Ouagadougou, Burkina Faso; e Rice Research Division, National Cereals Research Institute, Badeggi, Niger State, Nigeria; f Rice Research Station, Rokupr, PMB 736, Freetown, Sierra Leone; g Africa Rice Center (AfricaRice), C/o Central Agricultural Research Institute (CARI) Suakoko, Bong County, PMB 3929, Monrovia, Liberia; h Africa Rice Center (AfricaRice), P.O. Box 33581, Dar-es-Salaam, Tanzania

**Keywords:** High yield, New donors, Hotspots, West africa, Lowland rice

## Abstract

Iron (Fe) toxicity is recognized as one of the most widely spread soil constraints for rice production especially in West Africa. *Oryza glaberrima* the cultivated rice species that originated from West Africa is well-adapted to its growing ecologies. The aim of this study was to identify the promising *O. glaberrima* accessions tolerant to Fe toxicity from the 2106 accessions held at the AfricaRice gene bank. The screenings were conducted over a four-year period and involved evaluating the entries under Fe-toxic field conditions in West Africa, selecting good yielding accessions and repeating the testing with newly selected lines. Three accessions (TOG 7206, TOG 6218-B and TOG 7250-A) were higher yielding than *O. sativa* checks under stress but with similar yields under control conditions. These accessions yielded over 300 g/m^2^ under both Fe toxicity and control conditions. In conclusion, these materials could be used as donors in breeding programs for developing high yielding rice varieties suited to Fe toxicity affected areas in West Africa.

## 1 Introduction

*Oryza glaberrima* (genome AA, 2n = 24), the African rice, is one of the two cultivated rice species. It was the only rice species cultivated in Africa until the Portuguese introduced *O. sativa* from Asia (Linares, 2002). Since its introduction, *O. sativa* (Asian rice) has steadily replaced *O. glaberrima* and it is estimated that as of year 2000 <15% of the rice growing area was planted to *O. glaberrima* (WARDA, 1993, Linares, 2002). *O. sativa* is considered to be high-yielding and responsive to inputs but not well adapted to African conditions, while *O. glaberrima* is considered to be well adapted to African conditions but with a lower yield potential than *O. sativa* (Linares, 2002). Farmers continue to grow *O. glaberrima* in the harshest environments in West Africa (Jones et al., 1993).

*O. glaberrima* is considered as a rich reservoir of genes for tolerance to various biotic and abiotic stresses (WARDA, 1993, Jones et al., 1997, Sarla and Mallikarjuna Swamy, 2005, Futakuchi et al., 2012). However, in the literature most of the conclusions that are drawn about *O. glaberrima* species are based on studying only a few accessions. Although about 2500 *O. glaberrima* accessions are conserved at the Africa Rice Center (AfricaRice), intra-specific variation within *O. glaberrima* using the entire collection has rarely been investigated. There are only a few studies on genetic diversity based on field phenotyping (Jones et al., 1997, Ndjiondjop et al., 2012, Montcho et al., 2013). Furthermore, only a limited number of *O. glaberrima* accessions have been used in rice breeding programs. CG 14 and TOG 5681 are two *O. glaberrima* accessions which have been used as parents in developing New Rice for Africa (NERICA) varieties that have been adopted for cultivation in many countries in Africa (Somado et al., 2008, Sie, 2008). Lack of enough information on intraspecific variation within *O. glaberrima* is one of the main impediments to its use in breeding. Thus, it is important that the genetic variation within *O. glaberrima* for tolerance to various stresses are assessed and best-performing accessions be identified for use in breeding.

In Africa, rainfed lowlands occupy 40% of the total rice area (Seck et al., 2012). The average productivity of rainfed lowlands is quite low (1.5–2 t/ha) (GRiSP, 2013). One main reason for this low productivity is the prevalence of many stresses (Diagne et al., 2013, Saito et al., 2013). Among the several abiotic stresses affecting rice productivity, the most significant is Fe toxicity which is the most widely spread nutritional disorder in the lowlands of West Africa (Audebert, 2006, Sikirou et al., 2015). Up to 60% of the rice growing area is said to be affected by Fe toxicity in West African countries, resulting in an average yield loss of 50% (WARDA, 2002, Audebert and Fofana, 2009). Utilization of varieties with superior tolerance to Fe toxicity is the most economically viable option for resource limited farmers in affected areas. Sahrawat and Sika (2002) pointed out that *O. glaberrima* could be a good source of tolerance to Fe toxicity. However, only a limited number of accessions have been tested and only a few has been identified as tolerant, including CG 14 (Sahrawat and Sika, 2002) and TOG 5681 (Dramé et al., 2010). Further studies are required to fully exploit the genetic diversity for Fe toxicity tolerance in *O. glaberrima*.

In this study we systematically evaluated 2106 *O. glaberrima* accessions held by the AfricaRice gene bank for tolerance to Fe toxicity under field conditions in West Africa. The aim of this study was to identify, based on grain yield under stress, the most promising *O. glaberrima* accessions that could be used in breeding program for improved Fe toxicity tolerance.

## 2 Material and methods

### 2.1 Screening sites

Four Fe-toxic hotspots were selected in four different countries of West Africa, namely: Edozhigi in Nigeria, Valley du Kou in Burkina Faso, Suakoko in Liberia and Rokupr in Sierra Leone (Table 1). It has been reported that the Fe toxicity at these sites is so severe that susceptible rice varieties exhibit severe bronzing and completely die. These sites were characterized by high soil Fe-content (>295 mg kg^−1^) and acidic pH (pH 4.3–6.1) (Table 1). In addition to these four sites, a lowland rice field in IITA, Ibadan, Nigeria was used as the control site.

**Table 1:** Soil characteristics of the different sites used in this study.

Locations	Conditions	Texture	pH(H_2_O)	Fe Content	Latitude and Longitude	References
Edozhigi, Nigeria	Fe-toxic	clay loam	4.3	1230 mg kg^−1^	9°4′ 38.47 N 6°3′ 6.73” E	Abah et al. (2012)
Suakoko, Liberia	Fe-toxic	Sandy loam	5.2	489 mg kg^−1^	7°00′ 33” N 9°34′ 34” W	AfricaRice (unpublished data)
Valley du Kou, Burkina Faso	Fe-toxic	Silt loam	6.1	295 mg kg^−1^	11°11′ 30” N 4°18 36” W	Narteh and Sahrawat (1999)
Rokupr, Sierra Leone	Fe-toxic	Clay	5.7	548 mg kg^−1^	9°00′ 42” N 12°57′ 13” W	AfricaRice (unpublished data)
Ibadan, Nigeria	Control	Clay	7.0	84 mg kg^−1^	7°25′ 56” N 4°00′ 07” E	Ande (2014)

### 2.2 Plant materials

*O. glaberrima* accessions were obtained from the gene bank of the Africa Rice Center (AfricaRice, 2015) at Cotonou, Benin Republic. The set included the two *O. glaberrima* parents of NERICA varieties (CG 14 and TOG 5681). Two rice varieties popular in rainfed lowlands in Nigeria (WITA 4 and NERICA-L 19) were considered as checks and these are known to perform well under Fe toxicity conditions (Dramé et al., 2010, Sikirou et al., 2015). WITA 4 is an *O. sativa* variety while NERICA-L 19 is an interspecific derivative of a cross between IR 64 (*O. sativa*) and TOG 5681 (*O. glaberrima*).

### 2.3 Screening of *O. glaberrima* accessions

#### 2.3.1 Preliminary screening

The first evaluation was conducted in the wet season (WS) of 2011. The 2106 *O. glaberrima* accessions and checks were seeded in a nursery at Edozhigi, a Fe toxic hotspot in Niger state, Nigeria (Table 1). Out of the 2106 accessions, 1732 germinated and were transplanted in the main field as an un-replicated trial. The remaining 374 did not germinate due to either strong dormancy or loss of viability. The trial was laid out in 58 blocks with 30 plots (entries) in each block. At maturity stage, 253 accessions were selected based on visual yield assessment and only those accessions were harvested to determine actual grain yield. Accessions which had late flowering (>110 days after seeding), severe lodging (plant height above 160 cm) or severe shattering were eliminated prior to selection. As the number of entries was large and the experiment was un-replicated, selection was done within blocks to control for spatial variation. About five entries per block were selected and the 253 selected accessions were grouped into a set for further testing. Large ranges in trait values for grain yield (32–564 g/m^2^; average was 263 g/m^2^), days to flowering (67–102 days), plant height (83–160 cm) and tiller no. (4–26) were observed in this selected set. Among these, 206 accessions had higher yield than the best check (WITA 4; 135 g/m^2^). CG 14 was rejected due to its poor performance while TOG 5681 was selected.

#### 2.3.2 Replicated screening

The 253 accessions selected from previous study and standard checks were screened again in 2012 WS in Fe-toxic conditions at Edozhigi. Table 2 shows the geographical distribution of the 253 selected accessions of *O. glaberrima* used in this study (AfricaRice, 2015). Accessions with height above 150 cm and days to flowering of over 140 DAS were eliminated before selection in Edozhigi in 2012.

**Table 2:** Geographical origin of the *Oryza glaberrima* accessions selected for Fe toxicity tolerance.

Country of origin	Number of accessions tested per year		
	2012	2013	2014
Burkina-Faso	5	1	–
Chad	2	1	1
Côte d'Ivoire	9	2	–
Gambia	2	–	–
Ghana	3	–	–
Guinea	30	7	4
Guinea-Bissau	3	–	–
Liberia	75	5	1
Mali	66	18	8
Madagascar	1	–	–
Nigeria	37	7	2
Senegal	9	2	–
Sierra Leone	1	–	–
Togo	1	1	1
Unknown	9	3	1

Total	253	47	18

Based on grain yield in 2012 at Edozhigi, 44 best accessions were selected for multi-location trials and three standard checks were screened along four different Fe-toxic hotspots in four countries in West Africa in 2013 WS (Table 1). A control trial was conducted in Ibadan. Based on grain yield and leaf bronzing score (LBS), the 18 best accessions were chosen in Edozhigi in 2013. Accessions with days to flowering of over 125 DAS and plant height above 150 cm under stress were excluded from the selections.

The 18 accessions selected in 2013 were again re-evaluated in Edozhigi, Nigeria in the WS of 2014.

### 2.4 Experimental design and trial management

All the replicated trials were laid out in an alpha lattice with two replications except the 2014 trial in Edozhigi which had three replications. In all trials, plots comprised a single row of 3-m except in 2014 when two rows were planted. The distance between plots was 20 cm.

The seedlings were raised in a nursery and 21 day-old seedlings were transplanted. Two seedlings were transplanted per hill at a spacing of 20 cm between rows and between hills. NPK (15:15:15) compound fertilizer was applied at 200 kg/ha before transplanting. Two additional splits of urea were top-dressed each at the rate of 50 kg/ha at 42 days after seeding (DAS) and 56 DAS respectively. Approximately 5 cm of standing water was maintained in the field until harvest. The plots were weeded twice to avoid weed infestation.

### 2.5 Data collection

Crop characteristics such as leaf bronzing score (LBS), number of tillers per hill, days to flowering, plant height at maturity and grain yield per plot were collected at the appropriate growth stage of rice plant following the Standard Evaluation System (SES) of rice (IRRI, 2002). The leaf bronzing score was expressed on a 0–9 scale, (where 0 = normal or nearly normal plant; 9 = nearly dead or dead plant) and was scored at 80 days after seeding (DAS). Days to flowering was recorded when 50% of the plants in the plot started to flower. Plant height was measured at maturity as the average distance from the ground to the tip of the longest panicle of three plants randomly selected in each plot. Tiller number was recorded as the average number of tillers from three randomly selected hills in a plot. For each plot, panicles were harvested, dried, threshed and grains cleaned and weighed. Grain yield was calculated as the weight of filled grains per plot adjusted to 14% moisture content. Grain yield data were only collected from trials conducted in Edozhigi and Ibadan during 2012–2014. LBS and plant height were not recorded at Rokupr, Sierra Leone, while, days to flowering and plant height were not recorded at Valley du Kou, Burkina Faso.

### 2.6 Statistical analysis

Analysis of variance was done using REML option of the MIXED procedure of Genstat discovery edition 4th. Least squares means of accessions within location was generated and they were separated using LSD (P < 0.05). Genotypes were considered as fixed effects while replications and block within replications were considered as random effects. Variance components for each trial were estimated using the REML option of the VARCOMP procedure where all factors were considered to be random. Broad sense heritability (H) was computed from variance components as:

$$H=\frac{\sigma_{G}^{2}}{\sigma_{G}^{2}+\frac{\sigma_{E}^{2}}{r}}$$

where *σ^2^_G_* is the genetic variance, *σ^2^_E_* is the error variance, and *r* the number of replications.

Genetic correlations between two traits measured in the same environment within the same trial were calculated as follows:

$$r_{G12}=\frac{Cov_{12}}{\sqrt{\sigma_{G1}^{2}x\sigma_{G2}^{2}}}$$

(Bernado, 2010) where $r_{G12},$ $Cov_{12},$ $\sigma_{G1}^{2}$ and $\sigma_{G2}^{2}$ are the genetic correlation coefficient between traits 1 and 2 within the same trial, genetic covariance of traits 1 and 2, and the genotypic variances of traits 1 and 2, respectively.

## 3 Results

### 3.1 Performance of *O. glaberrima* accessions under Fe toxicity and control conditions

Highly significant differences among entries were observed for grain yield in both Fe-toxic (Edozhigi) and control (Ibadan) conditions in Nigeria during 2012–14 (Table 3). High heritability (H between 0.52 and 0.94) was observed both in stress and control trials. The mean grain yield of *O. glaberrima* accessions in stress trials was 233 g/m^2^ while under control conditions it was 298 g/m^2^ in 2013. In all the stress trials the best yielding *O. glaberrima* accession significantly out-yielded the best *O. sativa* check. However, under control conditions the yield of the best yielding accession of *O. glaberrima* was on par with the best *O. sativa* check. Grain yield of TOG 5681 was on par with *O. sativa* checks in both stress and control conditions. In the 2012 stress trial, six accessions significantly out-yielded the best *O. sativa* check (WITA 4; 248 g/m^2^), while in the 2013 stress trial only two accessions had significantly higher yield than the best check (WITA 4). In 2013 average grain yield under stress was lower than that in the control treatment by 23%. About 20% of the entries had a yield reduction of over 50% in the stress trial compared to the control trial and about 20% of the entries showed higher yield under stress than in the control (data not shown). Six accessions had higher yield than WITA 4 across the two trials in 2013. In 2014 trial four accessions significantly out-yield the best O. sativa check (NERICA-L 19).

**Table 3:** Grain yield of *Oryza glaberrima* accessions selected under Fe toxicity and checks under control and Fe-toxic conditions during 2012–14.

Genotype	Grain yield (g m^−2^)			
	Control	Fe Toxicity		
	Ibadan, Nigeria (2013 WS)	Edozhigi, Nigeria (2012 WS)	Edozhigi, Nigeria (2013 WS)	Edozhigi, Nigeria (2014 WS)
*O. glaberrima*				
Number of genotypes	44	253	44	18
Min	70	32	74	161
Max	520	544	464	430
Mean	298	213	233	277

Selections				
TOG 7206	495	428	302	430
TOG 14367	480	305	157	411
TOG 7250-A	360	468	433	365
TOG 6218-B	330	437	464	343

*Checks*				
WITA 4 (*O. sativa*)	450	248	263	294
NERICA-L 19 (Interspecific)	Na	228	131	304
TOG 5681 (*O. glaberrima*)	452	357	147	Na

P	<0.0001	<0.001	<0.01	<0.001
LSD (0.05)	145	204	169	20
Heritability	0.80	0.52	0.55	0.94

na not available.

Table 4 shows the leaf bronzing score of accessions at 80 DAS in five Fe-toxic sites. Significant differences (P < 0.001) were observed among accessions in Edozhigi during the WS 2014; in “Valley du Kou” and in Suakoko during the WS of 2013. Average heritability (H) across trials for LBS was about 0.5 and ranged from 0.11 to 0.73. Heritability was lower in the 2012 trial where the number of entries was higher. The mean LBS ranged from 2 to 5 across the trials. The LBS score of some *O. glaberrima* accessions were either higher, on par or lower than the LBS score of *O. sativa* checks. The LBS at Edozhigi varied from year to year (Table 4).

**Table 4:** Varietal means for leaf bronzing score of the *Oryza glaberrima* accessions selected under Fe toxicity and check varieties in three different Fe-toxic hotspots at 80 days after sowing during 2012–14.

Genotype	Leaf bronzing score				
	Edozhigi, Nigeria (2012 WS)	Edozhigi, Nigeria (2013 WS)	Valley du Kou, Burkina Faso (2013 WS)	Suakoko, Liberia (2013 WS)	Edozhigi, Nigeria (2014 WS)
*O. glaberrima*					
Number of genotypes	253	44	44	44	18
Min	3	1	2	3	1
Max	7	3	4	6	4
Mean	5	2	3	4	2

Selections					
TOG 7206	4	2	3	5	2
TOG 14367	5	2	1	2	1
TOG 7250-A	4	2	3	5	2
TOG 6218-B	4	2	1	4	1

*Checks*					
WITA 4 (*O. sativa*)	5	3	2	3	5
NERICA-L 19 (Interspecific)	5	3	3	3	4
TOG 5681 (*O. glaberrima*)	4	2	3	4	na

P	0.12	0.34	<0.001	<0.001	<0.001
LSD (0.05)	2	1	2	1	2
Heritability	0.11	0.32	0.60	0.73	0.67

na not available.

### 3.2 Performance of selections over sites

In the 2014 stress trial four entries (TOG 7206, TOG 14367, TOG 7250-A, TOG 6218-B) significantly out-yielded the checks (Table 3). These lines were selected and their performance was compared across trials in Edozhigi during 2012–14. The four selections yielded either significantly better or were on par with the *O. sativa* checks in all the stress and control trials. Similarly, the four selections either significantly out-yielded TOG 5681 or were on par with it in stress and control trials.

Three accessions (TOG 7206, TOG 7250-A and TOG 6218-B) out of the four have recorded consistently higher yield than the best check in stress trials in all the years. They showed both high yield potential and stress tolerance. The three accessions, TOG 7206, TOG 7250-A and TOG 6218-B were from Côte d’Ivoire, Burkina Faso and Mali respectively.

### 3.3 Genetic correlation

Genetic correlation between yield and other traits in three trials at Edozhigi (Fe toxic site) and one trial in Ibadan (control site) were estimated (Table 7). In Edozhigi in 2012, when number of entries was higher, grain yield and LBS was significantly negatively correlated. No such correlation was observed in 2013 and 2014 trials. Similarly, in Edozhigi in 2012 grain yield was significantly positively correlated with days to 50% flowering and plant height but no such correlation was observed in 2013 and 2014. At Ibadan (control) in 2013, grain yield was significantly negatively correlated to days to 50% flowering.

## 4 Discussion

In this study we systematically evaluated the *O. glaberrima* germplasm held at AfricaRice’s gene bank for Fe toxicity tolerance and identified high-yielding accessions that could be used in rice breeding programs. Out of 2106 accessions tested, we selected three promising accessions that can be used as parental materials for breeding for tolerance to Fe toxicity. This is the first study reporting a systematic and extensive screening of *O. glaberrima* accessions for Fe toxicity tolerance.

*O. glaberrima* is generally considered as low yielding compared to *O. sativa* (Linares, 2002, Guei et al., 2004). Its low yield potential is mainly due to low spikelet no., grain shattering, and lodging (WARDA, 1993, Jones and Singh, 1999, Montcho, 2013). One report states that in West African conditions, with *O. glaberrima*, yields of up to 5 t/ha are possible in irrigated fields (Futakuchi and Jones, 2005). In this study we observed that in all the yield trials, including in stress and irrigated conditions, the best accession always yielded above 400 g m^−2^ and at least in a few cases some *O. glaberrima* accessions yielded above 500 g m^2^ (Table 3). At least 10% of the *O. glaberrima* accessions in a trial yielded higher than the best *O. sativa* check (data not shown). The best yielding *O. glaberrima* accessions in each trial either significantly out-yielded the checks or were at least on par with them but never inferior. In two out of three years, under Fe stress, the best *O. glaberrima* accession yielded at least twice as much as the best *O. sativa* check (Table 3). This clearly demonstrates that *O. glaberrima* accessions are not necessarily lower yielding than *O. sativa* and that they possess higher Fe toxicity tolerance than some of the popular *O. sativa* cultivars.

*O. glaberrima* is considered to have a narrow genetic base (McCouch et al., 2013). Molecular analysis has revealed that genetic variability in *O. glaberrima* is lower than in *O. sativa* (Second, 1984, Wang et al., 1992, Ishii et al., 2001, Wang et al., 2014). In this study, we observed considerable phenotypic variations within *O. glaberrima* for most of the phenotypic traits measured. There was still significant variation in the final trial in Edozhigi, even with just 18 accessions.

We have strongly selected for higher yield under stress (Fe toxicity) and control conditions and lower LBS under stress. During the process we have also selected against strong dormancy, poor vigor and seed viability, high lodging, severe shattering, and strong photosensitivity (data not shown). At the main selection environment (Edozhigi, Nigeria), the average grain yield of *O. glaberrima* accessions increased from 213 to 277 g/m^2^ (Table 3). At the same time, LBS reduced from 5 to 2, days to flowering reduced from 108 days to 81 days and plant height reduced from 121 cm to 112 cm (Table 4, Table 5, Table 6). Three accessions TOG 7206, TOG 6218-B and TOG 7250-A were identified as consistently high-yielding under Fe toxicity (yielding between 300–468 g/m^2^; Table 3). The selected lines are shown to have high yield under irrigated conditions, as well (yielding between 330–500 g/m^2^; Table 3). TOG 7206 was the highest yielder in the final screening under stress during 2014 WS, it significantly out-yielding the other *O. glaberrima* selections and the *O. sativa* checks. TOG 7250-A significantly out-yielded the *O. sativa* checks under stress in three out of the four seasons. Similarly, TOG 6218-B significantly out-yielded the *O. sativa* checks under stress in two out of the four seasons and in 2013 season it out-yielded NERICA-L19 but not WITA 4. TOG 7250-A and TOG 6218-B significantly out-yielded TOG 5681 under stress in 2013 WS. The accessions in the last trial in Edozhigi in 2014 selected were similar to *O. sativa* checks in terms of days to flowering (79–96 days; Table 5) and plant height (111–123 cm; Table 6). Besides, they did not show lodging or strong dormancy in any of the trials. Shattering was not a problem if the plants were harvested on time (data not shown). Thus we can conclude that these accessions were not agronomically inferior. These accessions should be useful as donors in breeding for enhanced yield under Fe toxicity conditions. They can be used in interspecific breeding in combination with *O. sativa* or in intraspecific breeding with other *O. glaberrima* accessions. Alternatively, if found suitable upon extensive testing they can be recommended as varieties in the Fe-toxic areas where farmers are abandoning rice cultivation due to a lack of varietal options.

**Table 5:** Varietal means for days to flowering of the *Oryza glaberrima* accessions selected under Fe toxicity and check varieties in control and Fe-toxic conditions during 2012–14.

Genotype	Days to flowering					
	Control	Fe toxicity				
	Ibadan, Nigeria (2013 WS)	Edozhigi, Nigeria (2012 WS)	Edozhigi, Nigeria (2013 WS)	Suakoko, Liberia (2013 WS)	Rokupr, Sierra Leone (2013 WS)	Edozhigi, Nigeria (2014 WS)
*O. glaberrima*						
No. of genotypes	44	253	44	44	44	18
Min	87	89	72	106	84	72
Max	129	137	149	177	96	97
Mean	105	111	108	149	94	81

Selections						
TOG 7206	101	114	113	147	98	85
TOG 14367	122	129	125	176	96	96
TOG 7250-A	99	114	104	142	95	79
TOG 6218-B	100	112	104	133	91	80

*Checks*						
WITA 4 (*O. sativa*)	110	111	106	100	92	97
NERICA-L 19 (Interspecific)	100	104	96	103	80	90
TOG 5681 (*O. glaberrima*)	89	96	92	Na	89	Na

P	<0.0001	<0.001	<0.001	<0.001	<0.01	<0.0001
LSD (0.05)	4	4	13	31	9	2
Heritability	0.92	0.94	0.89	0.60	0.53	0.94

na not available.

**Table 6:** Varietal means for plant height of the *Oryza glaberrima* accessions selected under Fe toxicity and check varieties in control (Ibadan) and Fe-toxic conditions (Burkina Faso, Liberia, and Nigeria) during 2012–14.

Genotype	Plant height (cm)				
	Control	Fe toxicity			
	Ibadan, Nigeria (2013 WS)	Edozhigi, Nigeria (2012 WS)	Edozhigi, Nigeria (2013 WS)	Suakoko, Liberia (2013 WS)	Edozhigi, Nigeria (2014 WS)
*O. glaberrima*					
No. of genotypes	44	253	44	44	18
Min	124	88	107	101	93
Max	170	159	168	128	157
Mean	151	121	131	119	112

Selections					
TOG 7206	157	134	132	134	117
TOG 14367	164	139	131	122	123
TOG 7250-A	144	128	134	120	111
TOG 6218-B	149	123	143	121	111

*Checks*					
WITA 4 (*O. sativa*)	127	104	100	89	105
NERICA-L 19 (Interspecific)	152	131	129	106	121
TOG 5681 (*O. glaberrima*)	124	112	102	120	Na

P	<0.0001	<0.001	<0.001	<0.05	<0.001
LSD (0.05)	6	24	6	25	3
Heritability	0.91	0.57	0.93	0.42	0.94

na not available.

In *O. sativa* significant negative correlation between grain yield and leaf bronzing score (LBS) is generally observed (Sikirou et al., 2015). LBS is often considered as a good secondary trait in breeding for Fe toxicity tolerance. However, the level of correlation between the two traits in *O. sativa* depends on stress intensity, testing condition, and type and number of varieties used (Audebert and Fofana, 2009). Similarly, we found significant negative correlation between grain yield and LBS while evaluating a larger number of the *O. glaberrima* accessions under stress (Table 7). This correlation was not observed when evaluating a smaller but highly selected set.

**Table 7:** Genetic correlations between grain yield and others traits under Fe-toxic (Edozhigi, Nigeria) and control (Ibadan, Nigeria) conditions in three years.

	Grain yield			
	Fe toxicity			Control
Traits	Edozhigi, Nigeria (2012 WS)	Edozhigi, Nigeria (2013 WS)	Edozhigi, Nigeria (2014 WS)	Ibadan, Nigeria (2013 WS)
Days to flowering	0.32**	−0.16^ns^	0.13^ns^	−0.40**
Plant height	0.34**	0.02^ns^	−0.16^ns^	−0.02^ns^
Leaf bronzing score	−0.25**	0.01^ns^	−0.04^ns^	–

^ns^ not significant.

^*^significant at p < 0.05.

** Significant at p < 0.01.

One main challenge in using *O. glaberrima* in interspecific breeding with *O. sativa* is the problem of sterility (Pham and Bougerol, 1993, Ghesquiere et al., 1997). However, in many cases the sterility barrier can be overcome with backcrossing. Breeding lines can be used to significantly improve fertility while crossing the two species (Deng et al., 2010, Lorieux et al., 2013). The backcrossing approach was used in the development of lowland NERICA varieties (Sie, 2008); NERICA-L 19, a variety tolerant to Fe toxicity, is an interspecific line released in many West African countries. It is a BC_3_F_4_ line derived from IR64 (a Fe toxicity susceptible *O sativa* used as recurrent parent) and TOG 5681. Thus, a relatively small amount of introgression of *O. glaberrima* into *O. sativa* has generated a tolerant line. Use of the new *O. glaberrima* donors in breeding can potentially generate much more valuable lines. Potential returns from adoption of such stress tolerant varieties are huge in Africa (Jones et al., 2001).

## 5 Conclusion

In this study, significant genotypic variation for Fe toxicity tolerance was found within the *O. glaberrima* species. Three accessions with high yield potential and Fe toxicity tolerance that are stable across different environments were identified. These accessions TOG 7206, TOG 6218-B 6 and TOG 7250-A can be used in breeding Fe toxicity tolerant rice varieties.
